# rGO nanomaterial-mediated cancer targeting and photothermal therapy in a microfluidic co-culture platform

**DOI:** 10.1186/s40580-020-0220-3

**Published:** 2020-03-17

**Authors:** Seok Gyu Mun, Hyung Woo Choi, Jong Min Lee, Jae Hyun Lim, Jang Ho Ha, Min-Jung Kang, Eun-Joong Kim, Lifeng Kang, Bong Geun Chung

**Affiliations:** 1 Department of Biomedical Engineering, Sogang University, Seoul, Korea; 2 Research Center, Sogang University, Seoul, Korea; 3 Department of Mechanical Engineering, Sogang University, Seoul, Korea; 4 School of Pharmacy, University of Sydney, Sydney, NSW Australia

**Keywords:** Microfluidic co-culture platform, Reduced graphene oxide, Caner targeting, Photothermal therapy, rGO-PEG-FA nanomaterial

## Abstract

We developed the microfluidic co-culture platform to study photothermal therapy applications. We conjugated folic acid (FA) to target breast cancer cells using reduced graphene oxide (rGO)-based functional nanomaterials. To characterize the structure of rGO-based nanomaterials, we analyzed the molecular spectrum using UV–visible and Fourier-transform infrared spectroscopy (FT-IR). We demonstrated the effect of rGO-FA-based nanomaterials on photothermal therapy of breast cancer cells in the microfluidic co-culture platform. From the microfluidic co-culture platform with breast cancer cells and human umbilical vein endothelial cells (HUVECs), we observed that the viability of breast cancer cells treated with rGO-FA-based functional nanomaterials was significantly decreased after near-infrared (NIR) laser irradiation. Therefore, this microfluidic co-culture platform could be a potentially powerful tool for studying cancer cell targeting and photothermal therapy.

## Introduction

Metastatic breast cancer is the leading cause of cancer-related deaths among females worldwide. Metastasis generally starts with the invasion into neighboring tissues by cells originating from primary tumors. The invaded cells are towards blood vessels and disseminate via bloodstreams to distant organs [1]. Plenty of new therapies for metastatic breast cancer are under study and the treatment is improved. However, the establishment of successful therapies that can target the metastasis is still challenging. According to previous studies, a monoclonal antibody against vascular endothelial growth factor (VEGF), namely, bevacizumab, has been shown effective when advanced metastatic breast cancer patients were treated with paclitaxel or docetaxel [2, 3]. The matrix metalloproteinase (MMP) involved in breast tumor invasion and metastasis has been considered as a promising target. Clinical trials are under study with MMP inhibitors in combination with doxorubicin [4]. Triple-negative breast cancer (TNBC) represents the most aggressive and metastatic tumors with no effective targeted therapies. Sunitinib decreased tumor volumes in TNBC patient-derived xenograft model via suppression of angiogenesis [5]. Currently, a clinical trial showed that the cytotoxic effect of the conventional route with cisplatin and gemcitabine on patients was enhanced in combined administration of the poly [ADP-ribose] polymerase 1 (PARP1) inhibitor iniparib [6]. Meanwhile, the endothelial cells of the vascular microenvironment surrounding the tumors promote the invasive capability of the breast cancer cells. In several co-culture experimental systems of breast cancer cells and endothelial cells, the highly-invasive breast cancer cells have previously been reported to break down the endothelial barriers through the reduction of cell–cell adhesion molecule expressions, such as platelet endothelial cell adhesion molecule-1 (PECAM-1) and vascular endothelial cadherin (VE-cadherin) [7]. In addition, the chemokines (e.g., growth-regulated protein beta (Gro-β), interleukin 8 (IL-8)) produced by endothelial cells could stimulate invasiveness of breast cancer cells highly expressing c-x-c motif chemokine receptor 2 (CXCR2) [8]. These mechanism studies about the connection between cancer cells and endothelial cells provide insights for cancer targeting therapy. Although a number of studies have previously been carried out, the resistance and redundant pathways in targeted therapies of metastatic breast cancers need to be solved in various ways to prevent metastatic relapses.

To reduce the cytotoxic effect and enhance the anti-cancer capability against specific cell lines, the biocompatible nanomaterials based on drug delivery system (DDS) have widely been studied for cancer therapy applications. DDS has generally employed multi-functional nanoparticles which are composed of a hydrophobic core and hydrophilic shell [9]. For example, carbon nanotubes, quantum dots, and polymeric micelles, and mesoporous silica nanoparticles can load various hydrophobic anticancer drugs by hydrophobic interactions [10–13]. However, this chemotherapy commonly affects both normal cells [14, 15] and cancer cells [16]. Recently, newly emerging near-infrared (NIR) light-mediated phototherapy modalities, such as photothermal therapy (PTT) and photodynamic therapy (PDT), have extensively been explored as promising alternative therapeutic approaches [17–20]. The combination of these two modalities, such as chemo-photothermal or chemo-photodynamic therapy, can effectively overcome their drawbacks [15, 21]. To utilize this dual therapy, nanoparticles need to deliver the anticancer drugs with photothermal agents or photosensitizers, which can generate cytotoxic singlet oxygen by consuming tumor-dissolved oxygen [22–25]. Over the past decade, graphene and its derivatives, such as graphene oxide (GO) and reduced graphene oxide (rGO), have emerged as excellent multifunctional biomaterials for PTT applications due to facile synthesis, high water dispersibility, easily tunable surface functionalization, and good biocompatibility [26–28]. In particular, the carboxyl groups on the surface facilitate the functionalization with polymer or conjugation of targeting moiety as well as the high surface area enables it to load hydrophobic anticancer drugs or photosensitizers via π–π interaction [29–31]. To enhance the cancer therapeutic efficacy, a number of nanomaterials are required to reach the tumor sites and permeate through cancer cell membranes [32, 33]. For this strategy, the target ligands (e.g., IL-I3 peptide, cyclic arginine-glycine-aspartic acid (cRGD), hyaluronic acid, folic acid (FA), and antibodies), can be conjugated to the surface of nanomaterials, showing higher cellular uptake by receptor-mediated endocytosis and larger accumulation of nanomaterials in cancer cells [32–35]. Liu et al. [15] reported phenylboronic acid, which has proven to be an efficient target ligand for sialic acid-over expressed tumor cells, was conjugated to the borate-coordination-polymer-coated polydopamine nanoparticles for chemo-photothermal applications. In addition, Yao et al. [21] demonstrated that multi-stimuli-responsive gold nanorod was used for HER2/CD44 dual-targeted PTT-PDT therapy against breast cancer cells. The graphene oxide (GO)-gold nanomaterials were also loaded with anticancer drugs for enhanced chemo-photothermal therapy using DNA aptamer AS1411, which was reported to have specific binding affinity to nucleolin inside breast cancer cells [36]. Reduced graphene oxide (rGO) is a material that is widely used in the field of electrochemical sensor and the conjugation with FA can expand the range of the sensor applications.

To confirm the efficacy and safety of the nanomaterials that can be used in cancer therapy, it is important to mimic the microenvironment of cancer cells [37–40]. The microfluidic platform has extensively been used for cancer diagnosis [41–43] and therapy [44–46] applications, because it has various advantages, such as precise mimic of the microenvironment of cancer cells and rapid optimization of cancer diagnosis and therapy conditions. Although animal study has widely been used to investigate new drugs for optimizing therapy conditions, it has an ethical concern. Alternatively, drug screening can be achieved by using microfluidic platforms [47]. A combinatorial therapy of doxorubicin and aspirin has been conducted to treat breast cancer cells in the microfluidic platform [48]. Although this previous study helped to understand anti-inflammatory cancer therapy, the co-culture environment has not been considered. The co-culture system is of great importance, because it can mimic in vivo metastatic environments [49]. The hepatocyte and fibroblast have previously been co-cultured in the microfluidic platform [50]. The microcapsules in which cells were co-cultured were fabricated by using polyethylene glycol (PEG). The co-culture of the cells in the microcapsules enhanced the survival and function of the cells. Recently, a hydrogel-based microfluidic co-culture platform was developed to study photothermal therapy [51]. Breast cancer cells and glioblastoma were injected into the microfluidic platform to investigate the effect of gold nanorod-mediated photothermal therapy. Also, a multi-layer microfluidic platform was developed by combining two layers including microwells and microchannels to study the effect of photothermal therapy [52]. However, the previous studies only investigated the effect of photothermal therapy by nanomaterials without any co-culture system. In this study, we developed a simple microfluidic platform using a pipette to create the co-culture environment without any complicated system and mimic the injection of therapeutic agents. We further confirmed that rGO-FA-based nanomaterials enabled the specific targeting of breast cancer cells and further enhanced the photothermal therapy effect in the microfluidic co-culture platform.

## Materials and methods

### Fabrication of the microfluidic co-culture platform

The microfluidic co-culture platform was designed using AutoCAD (Autodesk, USA) and was fabricated by a two-step photolithography process [53–55]. To carry out two-step photolithography, the height of 150 μm cell culture microchannel was made using the SU-8 100 (Microchem Corp., USA) photoresist and the height of 20 μm bridge microchannel is made using SU-8 2025 (Microchem Corp., USA) photoresist. The detailed dimension of the microfluidic co-culture platform was shown in Additional file 1: Fig. S1. Polydimethylsiloxane (PDMS, Sylgard 184, Dow Corning Corp., USA) solution was poured on the silicon wafers. To cure the PDMS, the silicon wafer was placed into the oven at 80 °C for 1 h. After curing PDMS, it was peeled off from the silicon wafer and was subsequently bonded on the glass slides using oxygen plasma treatment (Femto Science, Korea). To sterilize the microfluidic co-culture platform, it was washed with 70% ethyl alcohol and was then exposed to a UV light for 30 min.

### Synthesis of rGO-PEG-FA nanomaterials

GO aqueous solution was purchased from Graphene Square, Inc., Korea. Monofunctional PEG (MW 5000 Da, PEG-NH_2_) and FA-PEG amine (FA-PEG-NH_2_) were provided by Nanocs, Inc. (New York, NY, USA). 1-ethyl-3-(3-dimethylaminopropyl) carbodiimide (EDC), N-Hydroxysuccinimide (NHS), Sodium hydroxide, chloroacetic acid were purchased from Sigma-Aldrich (St. Louis, MO, USA). Carboxylated GO was synthesized using a modified method as previously described [56]. Briefly, GO solution (1 mg/mL) was ultrasonicated for 1 h. NaOH (2.4 g) and chloroacetic acid (2 g) were added to GO solution and were then ultrasonicated for 3 h to convert the hydroxyl and epoxy groups on the GO nanosheets to carboxyl groups. Carboxylated GO was dialyzed against a molecular weight cut off (MWCO) 3,500 dialysis membrane in deionized water for 3 days to remove dispensable ions. Finally, the carboxylated GO was obtained by freeze-drying for 48 h. To obtain the GO-PEG-FA nanomaterials, 20 mg of carboxylated GO was dissolved in phosphate buffered saline (PBS, 10 mL, pH 5.8) and were subsequently ultrasonicated for 10 min. EDC (1 mmol) and NHS (1 mmol) were added into the carboxylated GO solution. After 1 h, FA-PEG-NH_2_ (10 mg) was added and was additionally stirred for 18 h. To remove unreacted FA-PEG-NH_2_, GO-PEG-FA was purified using cellulose membrane (MWCO 6–8 kDa) and was lyophilized for 48 h. As a control, PEG-NH_2_ was also conjugated into the carboxylate GO. Reduction process of GO-PEG-FA nanomaterials was carried out by treating with 0.05% v/v of hydrazine monohydrate (80%) followed by heating to 80 °C for 15 min [57]. The reduced GO-PEG-FA (rGO-PEG-FA) nanomaterials were dialyzed by a dialysis membrane against deionized water for 1 day and the final product was obtained by freeze-drying for 48 h (Additional file 1: Fig. S2).

### Characterization of rGO-PEG-FA nanomaterials

To observe the morphology and size of rGO-PEG-FA nanomaterials, transmission electron microscopy (TEM) was performed by a JEOL JEM-2100F (Tokyo, Japan) operating at an acceleration voltage of 200 kV. For the TEM measurement, the sample was prepared by placing drop of the sample solution of 1 mg/mL ethanol onto a 200-mesh copper grid coated with carbon. The chemical conjugation of PEG and PEG-FA to carboxylated GO was confirmed by Fourier transform Infrared spectroscopy (FT-IR, Nicolet 6700, Japan) using KBr pellets at room temperature in the range of 4000 ~ 400 cm^−1^ at a resolution of 4 cm^−1^. FA in the rGO-PEG-FA nanomaterials was confirmed by measuring the absorbance at 280 nm using UV–visible spectroscopy (UV 1800, Shimadzu, Japan). The surface charges of the carboxylated GO, rGO-PEG, and rGO-PEG-FA were determined by zeta potential measurements using a Zetasizer Nano Z (Malvern Instruments, Malvern, UK).

### Cell culture

MDA-MB-231, a breast cancer cell, was cultured in a cell culture dish with RPMI 1640 (Thermo Fisher Scientific, USA) medium containing 10% fetal bovine serum (FBS, Thermo Fisher Scientific, USA) and 1% penicillin–streptomycin (Thermo Fisher Scientific, USA). The human umbilical vein endothelial cells (HUVECs) were cultured in a cell culture dish coated with 2% gelatin, using endothelial cell growth medium-2 (EGM-2, Lonza, Switzerland) growth medium containing 10% FBS and 1% penicillin–streptomycin. Both breast cancer cells and HUVECs were incubated in an incubator (5% CO_2_, 37 °C). To evaluate the cytotoxicity of the rGO-PEG-FA nanomaterials, cells were seeded at a density of 1 × 10^4^ per well in a 96-well plate and were incubated with the cell culture medium in an incubator. After 24 h, the rGO-PEG-FA nanomaterials were treated at a concentration of 0, 10, 20, 30, and 40 μg/mL in an incubator. After 4 h, 10 μL of the cell count kit-8 (CCK-8) assay (Dojindo Laboratories, Japan) was treated and incubated for 1 h. After the CCK-8 solution treatment, the cell viability was calculated by measuring the absorbance at a wavelength of 595 nm using a microplate reader (EL800, Bio-Tek Instrument, USA). Cytotoxicity assays were performed three times in response to NIR laser irradiation.

### Photothermal therapy in the microfluidic co-culture platform

HUVECs were stained with carboxyfluorescein diacetate succinimidyl ester (CFSE, Thermo Fisher Scientific, USA). 2 × 10^5^ HUVECs seeded into the HUVEC microchannel and co-culture microchannel. After 1 day, breast cancer cells were stained with Far-red (Thermo Fisher Scientific, USA). 2 × 10^5^ cells/mL breast cancer cells loaded into cancer microchannel and co-culture microchannel, respectively. The culture medium was exchanged daily for the 3 days. After 1 day, rGO-PEG-FA nanomaterials were treated with the cells for 4 h. The cells were also stained with the live/dead assay and were then irradiated with NIR laser for 10 min. The images in response to NIR laser irradiation were obtained by a fluorescent microscope (IX73, Olympus, Japan).

### Cellular uptake analysis

To confirm the cellular uptake of breast cancer cells and HUVECs using rGO-PEG-FA nanomaterials, each cell was seeded on 8-well plates (Ibidi, Germany) at a density of 2 × 10^4^ and was subsequently incubated at 37 °C. After 24 h, rGO-PEG-FA nanomaterials were treated at a concentration of 30 μg/mL in an incubator. The cells were then stained with Alexa Fluor 594 phalloidin (Thermo Fisher Scientific, USA) and 4′,6-diamidino-2-phenylindole (DAPI, Thermo Fisher Scientific, USA). To confirm the cellular uptake in the microfluidic co-culture platform, 30 μg/mL rGO-PEG-FA nanomaterials were loaded into the left channel inlet of microfluidic co-culture platform and was subsequently incubated for 4 h to allow diffuse them to all three microchannels. Cellular uptake images of the microfluidic co-culture platform were obtained using confocal imaging microscope (LSM 710, Carl Zeiss, Germany).

### Immunostaining and cell viability analysis

For immunocytochemistry, the cells cultured in the microfluidic platforms were washed with phosphate-buffered saline (PBS, Thermo Fisher Scientific, USA) and were fixed with 4% paraformaldehyde for 30 min at room temperature. After washing with PBS, the cells were treated with 1% Triton X-100 (Sigma Aldrich, USA) for 15 min and were then washed with PBS. After treatment with 1% bovine serum albumin (BSA, Sigma Aldrich, USA), non-specific protein binding was washed with PBS. The cells were then stained with Alexa Fluor 594 phalloidin overnight and with DAPI for 30 min. The cell viability in a 96-well plate was evaluated by CCK-8 assay and was normalized in a control group without treatment with rGO-PEG-FA nanomaterials. Cell viability in the microfluidic co-culture platform was evaluated using a live/dead cytotoxicity kit (Thermo Fisher Scientific, USA). 2 mM Ethidium homodimer-1 (EthD-1) and 4 mM Calcein AM solution were added to 10 mL PBS and were mixed to obtain a solution of about 2 μM of EthD-1 and 4 μM of Calcein AM. This solution was introduced into a microfluidic co-culture platform to carry out live/dead assay. Live/dead staining results were evaluated using a fluorescent microscope and Image J (National Institute of Health, USA).

## Results and discussion

### Analysis of the microfluidic co-culture platform

**Fig. 1:**
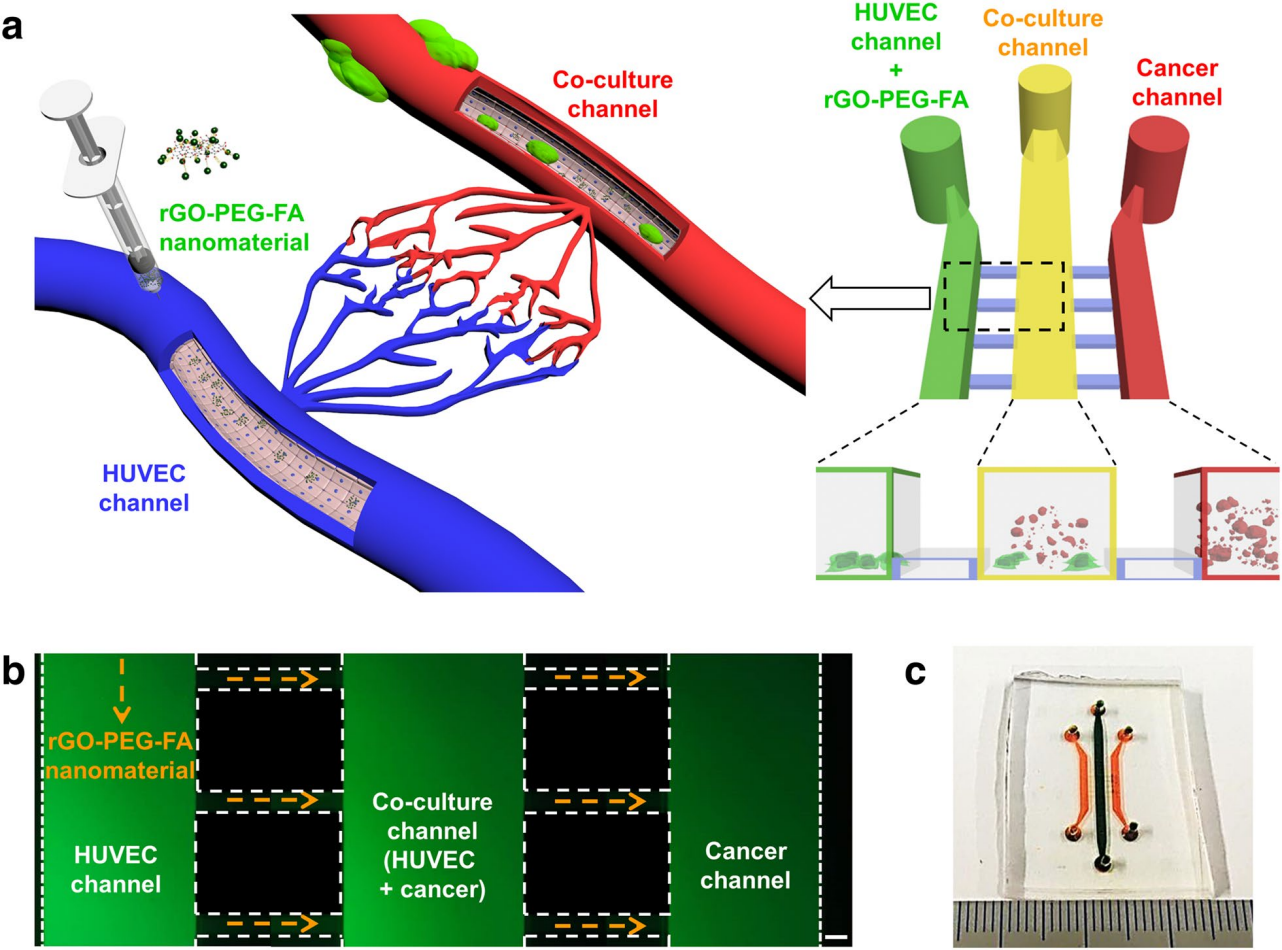
Schematic of the injection of therapeutic agents-mimicking microfluidic co-culture platform consisting of three cell culture channels and bridge channels (**a**). Microscope image of the microfluidic co-culture platform with green color dye (**b**). Photograph of the whole fabricated microfluidic co-culture platform (**c**). Scale bars are 50 μm

We fabricated the microfluidic co-culture platform using a two-step photolithography technique as previously described [53–55]. The microfluidic co-culture platform consisted of three cell culture channels (150 μm thickness) and bridge channels (20 μm thickness). The height difference between cell culture and bridge channels allowed for high fluidic resistance to minimize the cross-fusion of different cell types. Our microfluidic co-culture platform was designed to mimic the injection of therapeutic agents (Fig. 1a). The left HUVEC channel was used to simulate the injection of therapeutic agents via the blood vessels, the middle co-culture channel was used to simulate the metastatic breast cancer tissues by co-culturing of HUVECs and MDA-MB-231 cells. The right cancer channel was used to mimic primary carcinoma environment. In addition, the bridge channel was used to mimic capillaries connecting to each type of tissue. After seeding HUVECs and MDA-MB-231 breast cells in each microchannel, the functional rGO-PEG-FA nanomaterial was injected into the left HUVEC channel and the photothermal therapy effect was analyzed by NIR laser irradiation after diffusion of the rGO-PEG-FA nanomaterials (Fig. 1c). To co-culture cells in the microfluidic platform, a number of studies have previously been reported by using an external substance (e.g., hydrogel [58, 59]) or additional structure, such as microvalve [60] and micropillar array [61]. As compared to previous co-culture devices, we developed the simple microfluidic platform for co-culturing cells using a pipette without any complex structures. In the process of seeding HUVECs into the channel, a pipette tip was inserted into the inlet and outlet of the cancer channel to create a pressure difference. Using this process, HUVECs did not move to the right cancer channel, showing that HUVECS were spatially isolated in left HUVEC and middle co-culture channel. After 24 h, MDA-MB-231 breast cancer cells were seeded into the right cancer channel in the same manner and a pipette tip was inserted into the inlet and outlet of the HUVEC channel to form a pressure difference. This simple cell injection process enabled the spatial isolation of the cells in each channel without additional aids.

### Synthesis and characterization of rGO-PEG-FA nanomaterials

**Fig. 2:**
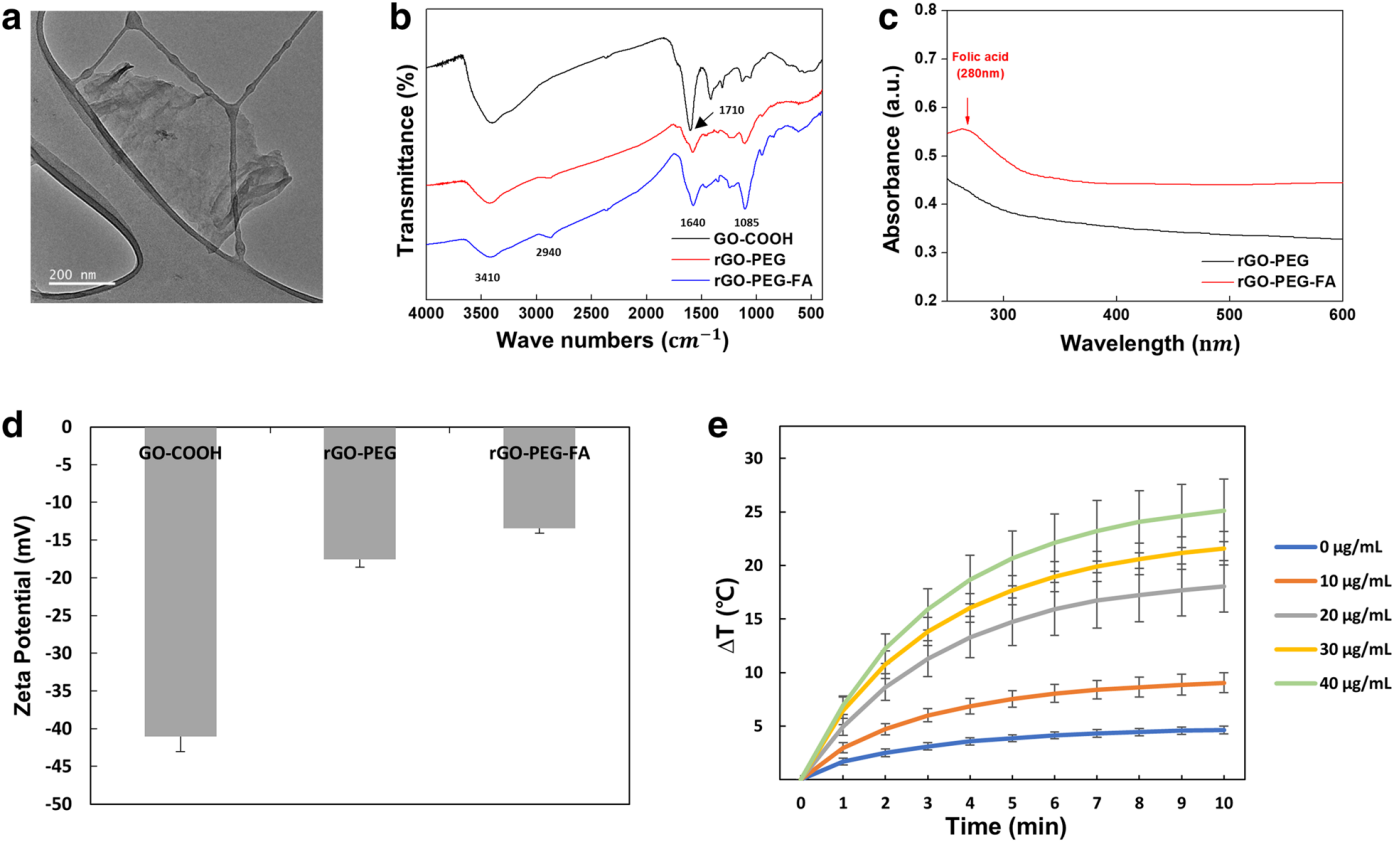
Characterization of nanomaterials. TEM image of rGO-PEG-FA nanomaterials (**a**). Scale bars are 200 μm. FT-IR spectra of GO-COOH, rGO-PEG, and rGO-PEG-FA nanomaterials (**b**). UV–visible spectra rGO-PEG, and rGO-PEG-FA nanomaterials (**c**). Zeta potential analysis of GO-COOH, rGO-PEG, and rGO-PEG-FA nanomaterials (**d**). Temperature analysis against concentrations of rGO-PEG-FA nanomaterials (**e**)

To observe the photothermal effect using nanomaterials in specific cancer cells under microfluidic co-culture platform, we synthesized rGO-PEG-FA nanomaterials and evaluated their physical and chemical characteristics. We prepared the carboxylated GO and PEG-FA was subsequently conjugated to carboxylated GO to enhance the photothermal effect and cancer targeting efficiency. TEM results revealed that rGO-PEG-FA nanomaterials (100 ~ 150 nm size) showed a spherical shape (Fig. 2a). The successful conjugation between the carboxyl group of GO and amino group of PEG/PEG-FA via EDC-NHS reaction was confirmed by FT-IR spectroscopy (Fig. 2b). Carboxylated GO showed the broad band around 3361 cm^−1^ corresponded to O–H stretching vibrations [56]. The characteristic stretching adsorption band originating from carbonyl groups (C=O) in the COOH units was observed at 1728 cm^−1^, indicating the introduction of carboxyl groups on the GO nanosheets. After conjugation of PEG on carboxylated GO, the characteristic peaks of PEG were observed at 1466 cm^−1^ and 1340 cm^−1^ due to the -CH_2_ and CH_3_ framework stretching in PEG. It also showed –C–O–C– asymmetrical and symmetrical stretching at 1097 cm^−1^ and 960 cm^−1^ [62]. Furthermore, the broad band around 3361 cm^−1^ corresponded to O–H stretching vibrations and this absorption was diminished in rGO-PEG, indicating that the chemical reduction of GO-PEG-FA was successfully performed. However, FA was not detected by FT-IR, because its structure was similar to PEG. To confirm the presence of FA for targeting breast cancer cells, rGO-PEG and rGO-PEG-FA nanomaterials were performed by UV–visible spectroscopy (Fig. 2c). While the spectrum of rGO-PEG did not show any absorption peak, rGO-PEG-FA showed the additional peak at 280 nm, which was distinctive in FA [63, 64]. This result supported that FA molecules were grafted on rGO surface with PEG. The zeta potential measurements were conducted to further verify the conjugation of PEG/PEG-FA on the rGO nanosheets (Fig. 2d). The carboxylated GO showed a negative surface charge (− 41 mV) and this value is lower than that of pure GO (− 35 mV) due to the presence of increased carboxyl groups. After conjugation of PEG on the rGO surface, the zeta potential decreased to − 17.5 mV. rGO-conjugated PEG-FA exhibited similar zeta potential values (− 13.4 mV). These changes of zeta potential are attributed to PEG molecules containing a number of amines [65], showing successful synthesis of rGO-PEG and rGO-PEG-FA nanomaterials. To demonstrate the photothermal effect of rGO-PEG-FA nanomaterials, the temperature change under NIR laser irradiation was monitored. rGO-PEG-FA aqueous solutions containing various concentrations (0 ~ 40 µg/mL) were exposed to an 808 nm NIR laser at a power density of 1 W/cm^2^ for 10 min. We confirmed that the photothermal effects of rGO-PEG-FA nanomaterials were dependent on concentrations (Fig. 2e). When 40 µg/mL concentration of rGO-PEG-FA nanomaterials was used, the temperature was elevated up to 25 °C. As a control, the temperature of pure water showed no obvious change. To investigate the photo-stability of rGO-PEG-FA nanomaterials, the temperature was recorded by three repeated cycles of NIR laser irradiation, suggesting that rGO-PEG-FA nanomaterials with low concentrations could be potential for photothermal therapy against breast cancer cells.

### Photothermal therapy effects in microfluidic co-culture platform

**Fig. 3:**
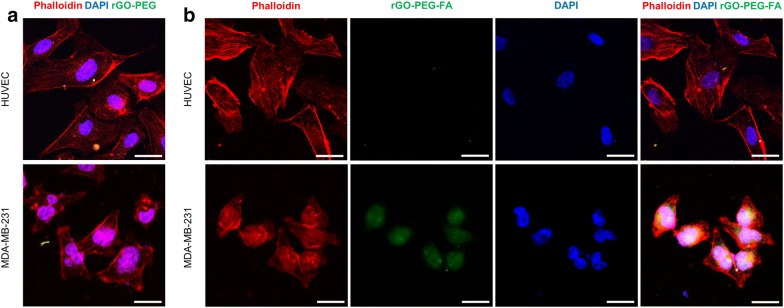
Confocal laser scanning microscopy images of HUVECs and MDA-MB-231 breast cancer cells treated with 30 μg/mL of rGO-PEG (**a**). Confocal laser scanning microscopy images of cells treated with 30 μg/mL of FA-conjugated rGO-PEG for targeting of breast cancer cells (**b**). Scale bars are 20 μm

**Fig. 4:**
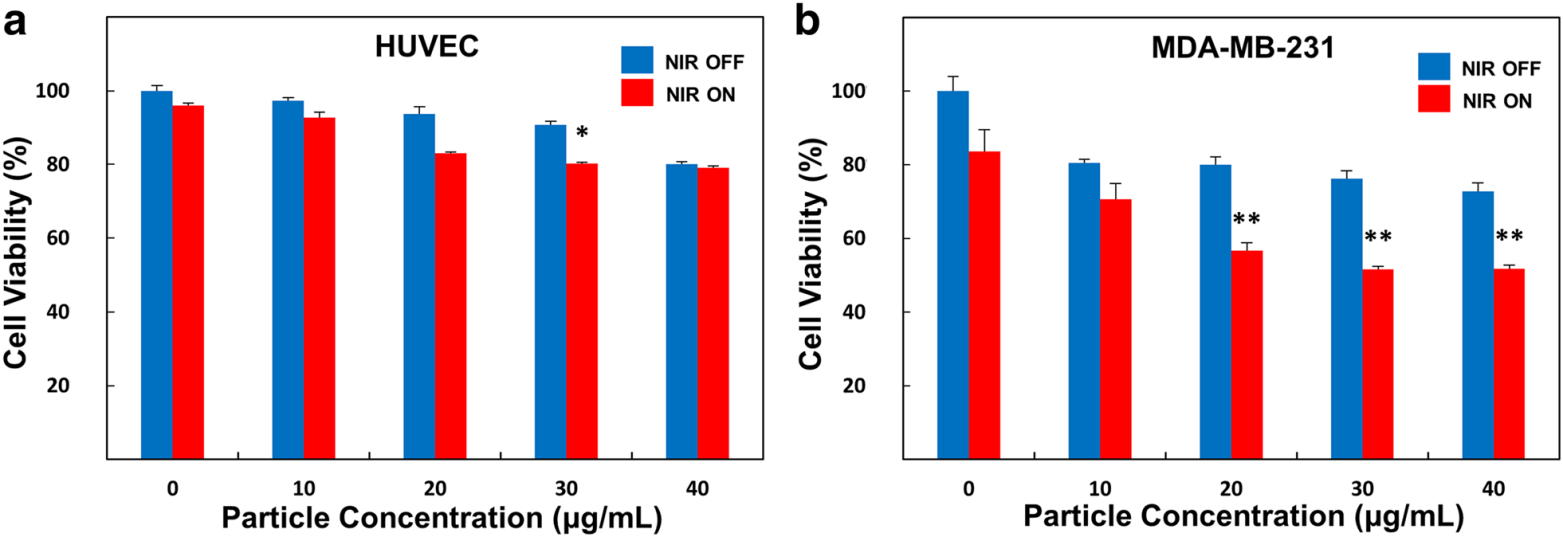
Analysis of cell viability. Evaluation of cytotoxicity of rGO-PEG-FA nanomaterials in HUVECs (**a**) and MDA-MB-231 breast cancer cells (**b**) before and after NIR laser treatment (*$p$ < 0.05, **$p$ < 0.01)

**Fig. 5:**
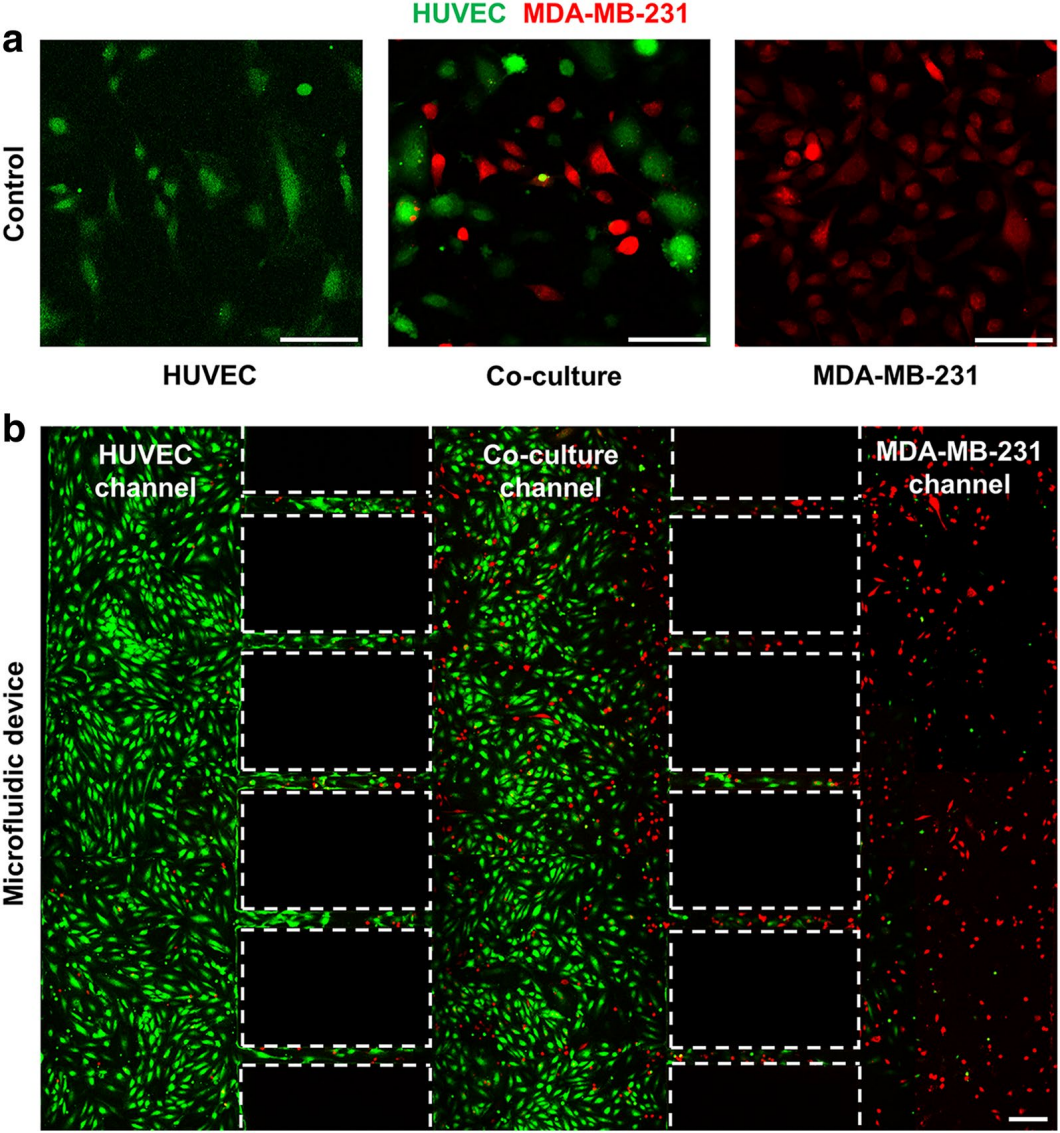
Confocal laser scanning microscopy images of HUVECs (green) and MDA-MB-231breast cancer cells (red) in a culture dish (**a**), in a microfluidic co-culture platform (**b**). Scale bars are 100 μm

**Fig. 6:**
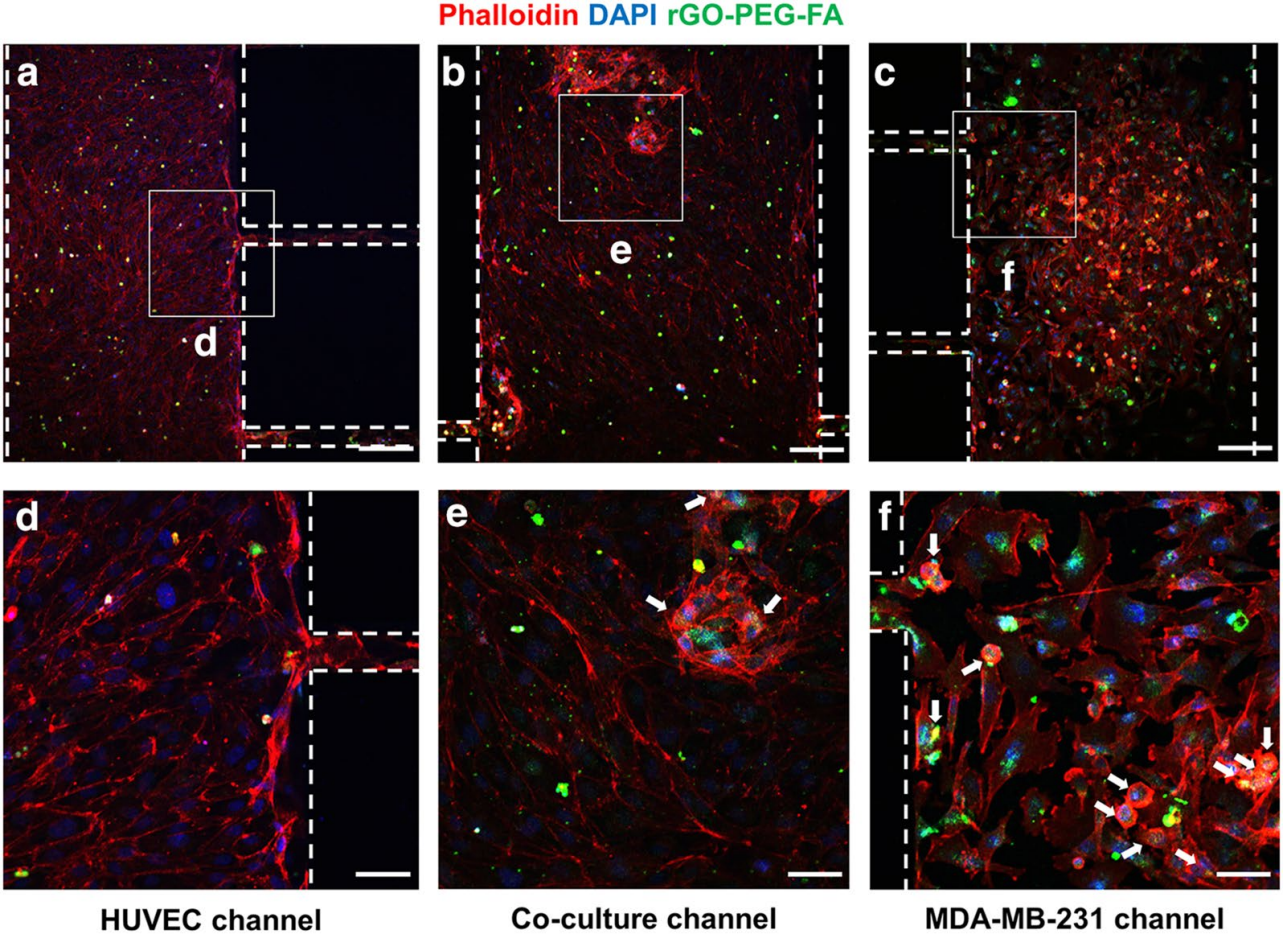
Confocal laser scanning microscopy images of cellular uptake after treatment with 30 μg/mL of rGO-PEG-FA nanomaterials in a microfluidic co-culture platform. Images of the HUVEC (**a**), Co-culture (**b**), and MDA-MB-231 (**c**) channel. High magnification images (**d**–**f**). White arrows indicate nanomaterial uptake in cells. Scale bars are 100 μm

**Fig. 7:**
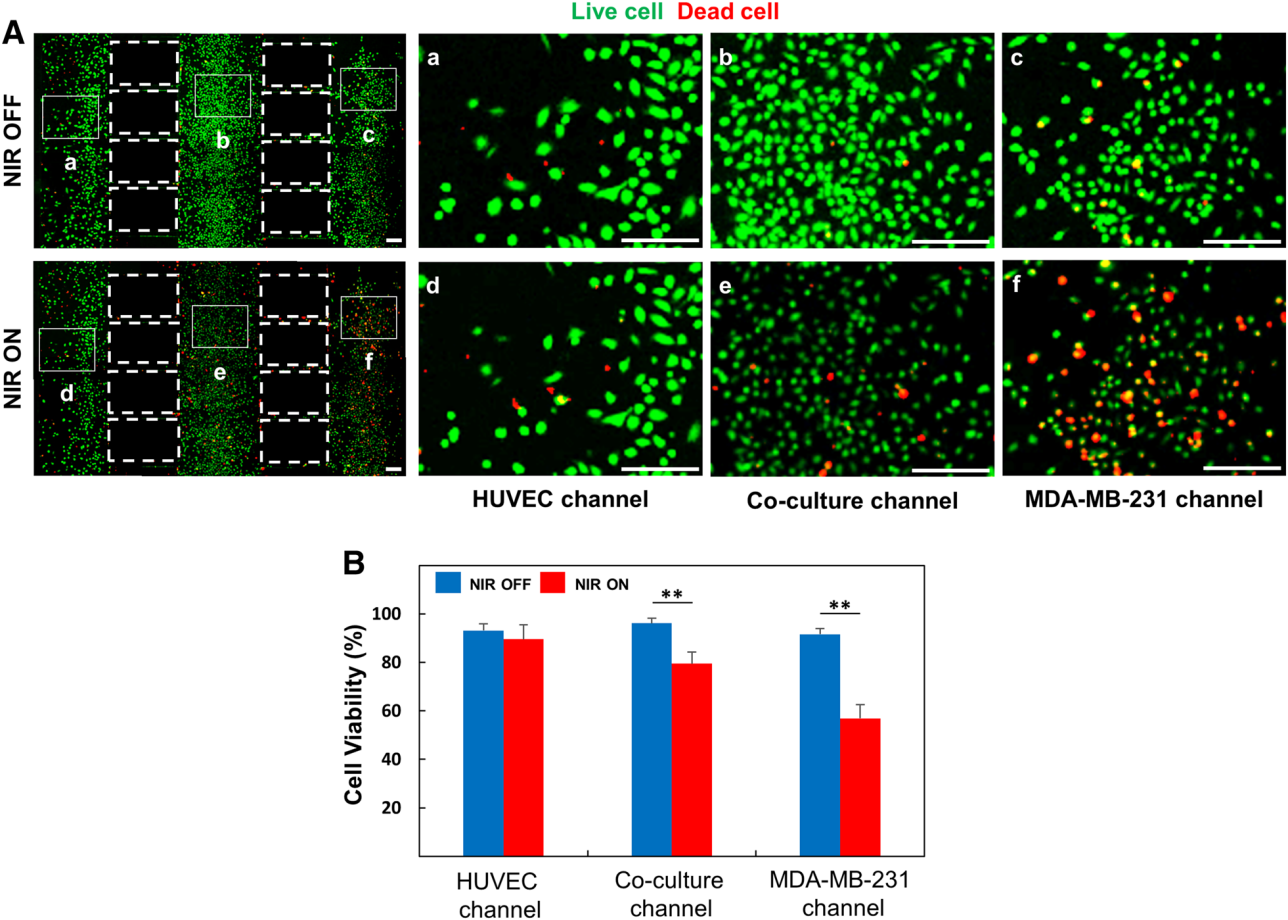
Fluorescence image of live (green)/dead (red) before and after NIR laser irradiation after treatment with 30 μg/mL of rGO-PEG-FA nanomaterials in HUVECs and MDA-MB-231 breast cancer cells (**A**). High magnification image of the HUVEC (a, d), Co-culture (b, e), and MDA-MB-231 (c, f) channel. Analysis of cell viability by fluorescence intensity in a microfluidic co-culture platform (**B**) (*$p$ < 0.05, **$p$ < 0.01). Scale bars are 100 μm

To confirm the cellular uptake of functional nanomaterials, rGO-PEG and rGO-PEG-FA nanomaterials treated to HUVECs and MDA-MB-231 cells (Fig. 3). FITC was conjugated to rGO-PEG and rGO-PEG-FA nanomaterials, respectively and endocytosis was subsequently confirmed by a laser scanning confocal microscopy. In rGO-PEG nanomaterials, the cellular uptake did not occur in HUVECs and MDA-MB-231 cells, because FA was not used (Fig. 3a). On the other hand, in rGO-PEG-FA nanomaterials, we observed that most of the rGO-PEG-FA nanomaterials were accumulated in the cytosol of the MDA-MB-231 breast cancer cells. In contrast, uptake of rGO-PEG-FA nanomaterials could not be observed in the HUVECs (Fig. 3b), indicating that HUVECs were negative for the folate receptor [66]. Folate receptors are highly overexpressed on the surface of many cancer cells including breast, lung, ovarian, brain, and colorectal cancer cells [67, 68]. Folate receptors bind FA and it has been extensively studied as molecular targets for delivery into cancer cells [69]. Hence, FA-modified rGO-PEG may be used to target MDA-MB-231 cells. The toxicity and photothermal effect of rGO-PEG-FA nanomaterials in HUVECs and MD-MB-231 cells were evaluated by CCK-8 assay (Fig. 4). Graphene-based nanomaterials are effective materials for photothermal therapy. First, it is essential to evaluate the toxicity of nanoparticles to the cells, as previously described [70, 71]. We observed that the cell viability of more than 91% was observed for HUVECs up to 30 μg/mL rGO-PEG-FA nanomaterials. In contrast, it was significantly decreased to 80% at 40 μg/mL rGO-PEG-FA nanomaterials, showing that is toxic at higher concentration (> 40 μg/mL) (Fig. 4a). In addition, the cell viability was decreased when rGO-PEG-FA nanomaterials were treated with MDA-MB-231 cells due to endocytosis of rGO-PEG-FA in cancer cells, as previously described [66, 68, 69]. To confirm the photothermal effect of rGO-PEG-FA nanomaterials, an 808 nm NIR laser was irradiated with an intensity of 2 W/cm^2^ for 10 min. In MDA-MB-231 breast cancer cells, 76% cell viability at a 30 μg/mL concentration of rGO-PEG-FA nanomaterials was decreased to 52% after NIR laser irradiation (Fig. 4b). Also, the cell viability was 52% at 40 μg/mL high concentration of rGO-PEG-FA nanomaterial. This result indicates that the cellular uptake of the nanoparticles is saturated [72, 73]. Therefore, we optimized at 30 μg/mL concentration of rGO-PEG-FA nanomaterials to apply photothermal therapy. To confirm the morphology of HUVECs and MDA-MB-231 breast cancer cells, HUVECs were stained with CFSE proliferation kit and MDA-MB-23 breast cancer cells were stained with Far Red kit (Fig. 5a, b). After culturing for 3 days, each cell was observed in the microfluidic co-culture platform, showing that there was no morphological difference between control and microfluidic co-culture platform. We also investigated the cellular uptake using rGO-PEG-FA nanomaterials in the microfluidic co-culture platform (Fig. 6). The fluorescent images displayed low (Fig. 6a–c) and high (Fig. 6d–f) magnification images in the microfluidic co-culture platform. We confirmed that the tendency of cellular uptake was similar to control (Fig. 3b). In the HUVEC channel, FITC fluorescence was not observed in the cytosol (Fig. 6a, d). In contrast, in the MDA-MB-231 channel, FITC fluorescence was observed in the cytosol (Fig. 6c, f). Interestingly, FITC fluorescence in the cytosol of MDA-MB-231 cells was selectively observed in the co-culture channel (Fig. 6b, e). Thus, it was confirmed that our microfluidic co-culture platform could simulate the injection of therapeutic agents through the diffusion of rGO-PEG-FA nanomaterials. After rGO-PEG-FA nanomaterials were treated in the microfluidic co-culture platform, the cell viability was analyzed using NIR laser irradiation (Fig. 7). The cell viability was confirmed by a live/dead assay (Fig. 7b). Cell viability in HUVEC, co-culture, and breast cancer channel was 93%, 96%, and 92%, respectively, before NIR laser irradiation. On the other hand, after NIR laser irradiation, 90%, 79%, and 57% were distinctly observed in each channel. The cell viability of the HUVECs was almost similar (90 ~ 93%) regardless of NIR laser irradiation. In the breast cancer channel, the cell viability in response to NIR laser irradiation was 92% and 57%, respectively. The cell viability in the co-culture channel was 96% and 79%, respectively. The presence of MDA-MB-231 breast cancer cells in the microchannel revealed the difference of cell viability in HUVEC channel. It is probably due to rGO-PEG-FA nanomaterial-mediated selective targeting of MDA-MB-231 breast cancer cells with folate receptors.

## Conclusions

We demonstrated that rGO-FA-based nanomaterials enabled the specific targeting of breast cancer cells with enhanced photothermal therapy effect in the microfluidic co-culture platform. Our microfluidic co-culture platform shows several advantages; (1) it enables the spatial isolation of two different types of the cells without any use of external substances (e.g., hydrogel), (2) it is possible to mimic the injection of therapeutic agents-based cancer therapy, showing that rGO-PEG-FA nanomaterials were loaded through the HUVEC channel and were gradually diffused into the co-culture and cancer channels through the bridge channel, and (3) rGO-PEG-FA nanomaterials enable the active targeting of breast cancer cells and significantly enhance the photothermal therapy effect. Therefore, our microfluidic co-culture platform could provide a powerful method for rGO-PEG-FA nanomaterial-mediated breast cancer targeting and photothermal therapy applications.

## Supplementary information

**Additional file 1: Figure S1.** Microfluidic co-culture platform with detail dimensions. **Figure S2.** Schematic synthesis process of rGO-PEG-FA nanomaterials.

## Data Availability

The authors have no data to share since all data are shown in the submitted manuscript.

## Abbreviations

rGO: Reduced graphene oxide
PEG: Polyethylene glycol
FA: Folic acid
HUVECs: Human umbilical vein endothelial cells
NIR: Near-infrared
PTT: Photothermal therapy
