# Supplementary material for: rGO nanomaterial-mediated cancer targeting and photothermal therapy in a microfluidic co-culture platform
Source: Nano Converg. 2020 Mar 17;7:10. doi: 10.1186/s40580-020-0220-3 (PMC7076105; doi:10.1186/s40580-020-0220-3)
Supplement: Supplementary file 1 — Additional file 1: Figure S1. Microfluidic co-culture platform with detail dimensions. Figure S2. Schematic synthesis process of rGO-PEG-FA nanomaterials. [file 40580_2020_220_MOESM1_ESM.docx]

**Additional file**

**rGO nanomaterial-mediated cancer targeting and photothermal therapy in a microfluidic co-culture platform**

Seok Gyu Mun^†,1^, Hyung Woo Choi^†,2^, Jong Min Lee^†,3^, Jae Hyun Lim^1^, Jang Ho Ha^3^,

Min Jung Kang^2^, Eun-Joong Kim^2^, Lifeng Kang^4^, Bong Geun Chung^3,*^

^1^ Department of Biomedical Engineering, Sogang University, Seoul, Korea

^2^ Research Center, Sogang University, Seoul, Korea

^3^ Department of Mechanical Engineering, Sogang University, Seoul, Korea

^4^ School of Pharmacy, University of Sydney, NSW, Australia

^†^These authors equally contributed to this work


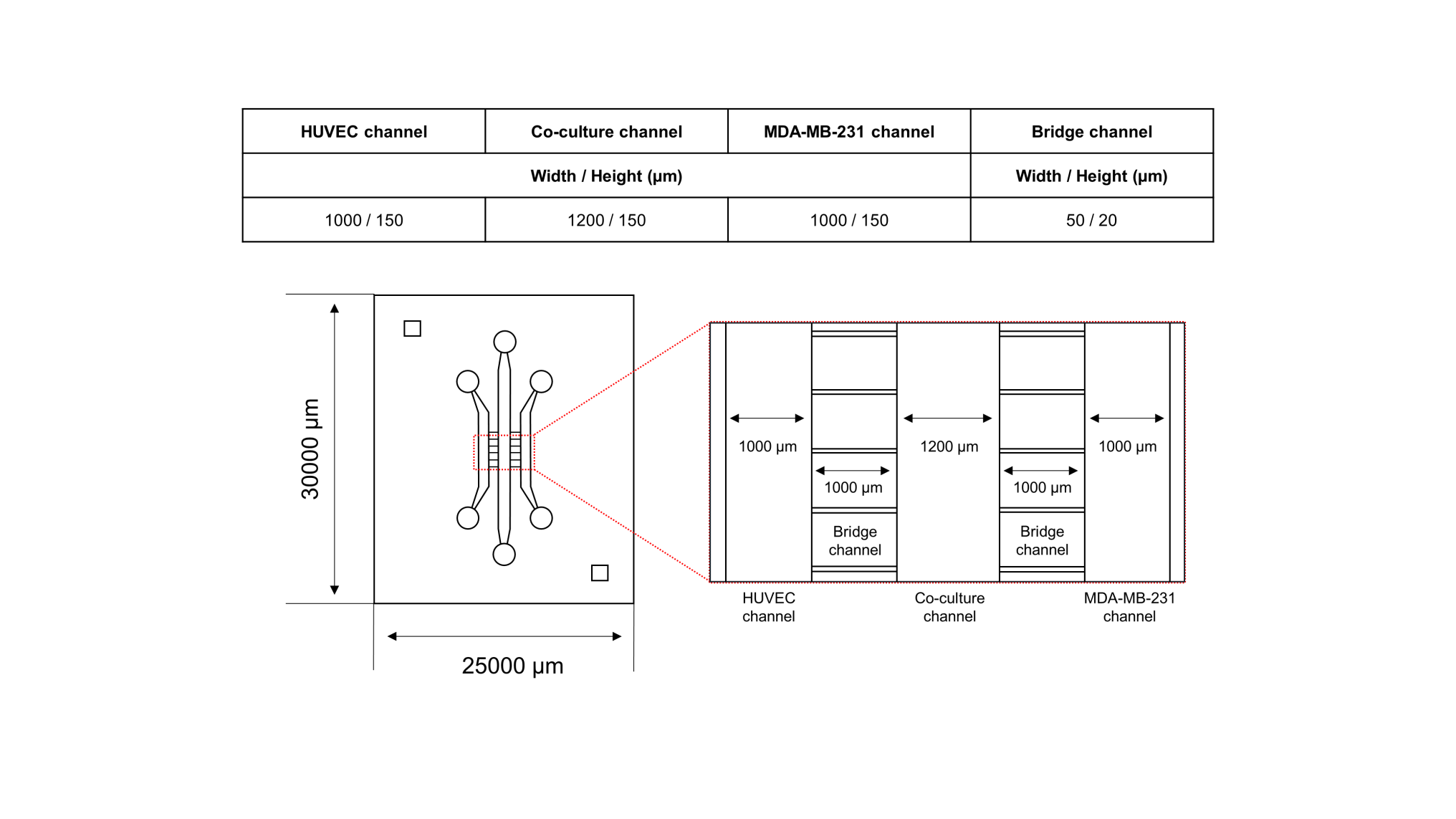


Figure S1**.** Microfluidic co-culture platform with detail dimensions.


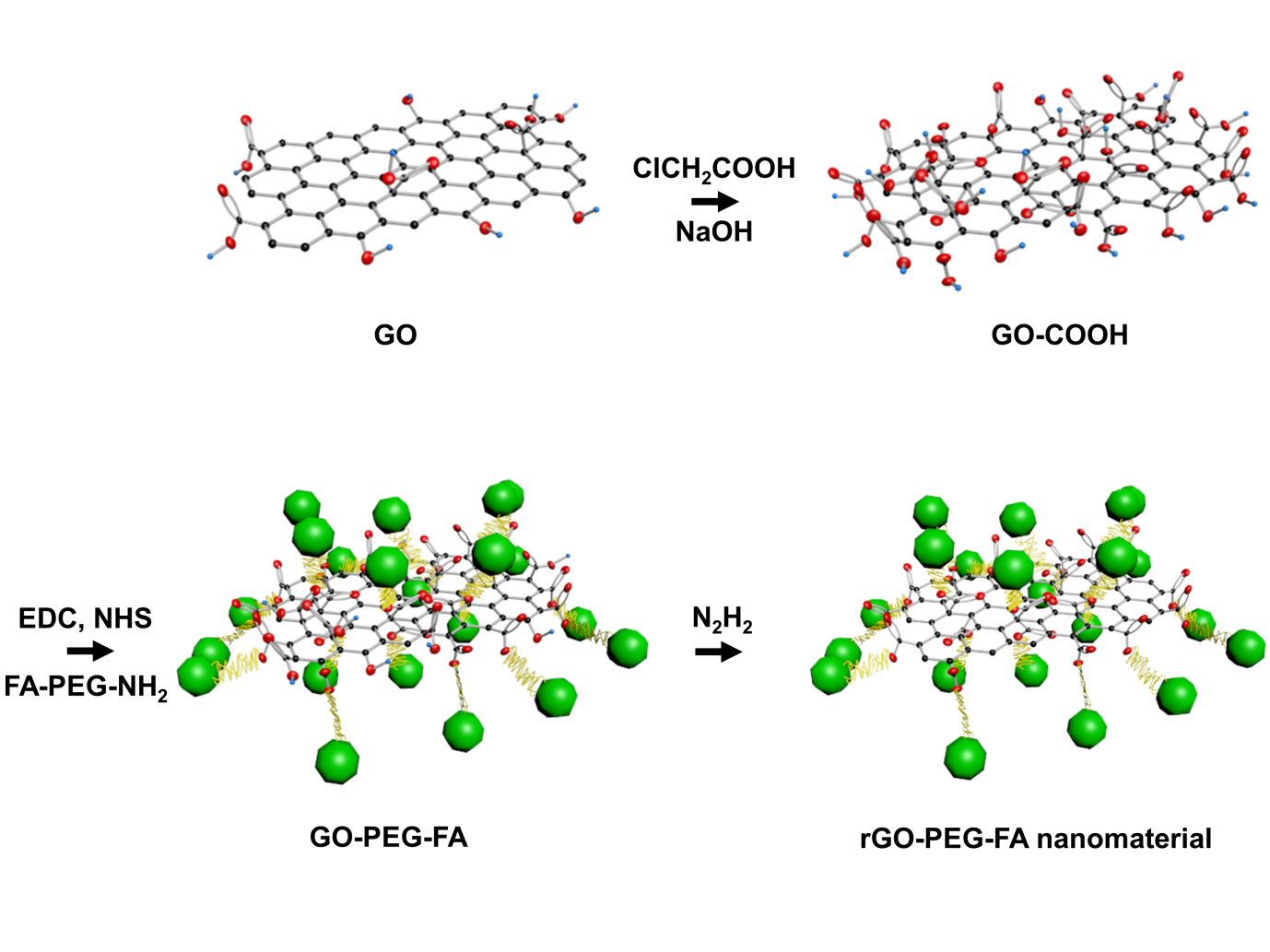


Figure S2. Schematic synthesis process of rGO-PEG-FA nanomaterials.
